# Emergency department quality and safety indicators in resource-limited settings: an environmental survey

**DOI:** 10.1186/s12245-015-0088-x

**Published:** 2015-10-31

**Authors:** Emily L. Aaronson, Regan H. Marsh, Moytrayee Guha, Jeremiah D. Schuur, Shada A. Rouhani

**Affiliations:** Department of Emergency Medicine, Brigham and Women’s Hospital, Boston, MA USA; Harvard Affiliated Emergency Medicine Residency, Brigham and Women’s Hospital/Massachusetts General Hospital, Boston, MA USA; Department of Emergency Medicine, Harvard Medical School, Boston, MA USA; Center for Clinical Excellence, Brigham and Women’s Hospital, Boston, MA USA; Department of Emergency Medicine, Massachusetts General Hospital, Boston, MA USA; Massachusetts General Hospital Division of Global Health and Human Rights, Boston, MA USA

## Abstract

**Background:**

As global emergency care grows, practical and effective performance measures are needed to ensure high quality care. Our objective was to systematically catalog and classify metrics that have been used to measure the quality of emergency care in resource-limited settings.

**Methods:**

We searched MEDLINE, Embase, CINAHL, and the gray literature using standardized terms. The references of included articles were also reviewed. Two researchers screened titles and abstracts for relevance; full text was then reviewed by three researchers. A structured data extraction tool was used to identify and classify metrics into one of six Institute of Medicine (IOM) quality domains (safe, timely, efficient, effective, equitable, patient-centered) and one of three of Donabedian’s structure/process/outcome categories. A fourth expert reviewer blinded to the initial classifications re-classified all indicators, with a weighted kappa of 0.89.

**Results:**

A total of 1705 articles were screened, 95 received full text review, and 34 met inclusion criteria. One hundred eighty unique metrics were identified, predominantly process (57 %) and structure measures (27 %); 16 % of metrics were related to outcomes. Most metrics evaluated the effectiveness (52 %) and timeliness (28 %) of care, with few addressing the patient centeredness (11 %), safety (4 %), resource-efficiency (3 %), or equitability (1 %) of care.

**Conclusions:**

The published quality metrics in emergency care in resource-limited settings primarily focus on the effectiveness and timeliness of care. As global emergency care is built and strengthened, outcome-based measures and those focused on the safety, efficiency, and equitability of care need to be developed and studied to improve quality of care and resource utilization.

**Electronic supplementary material:**

The online version of this article (doi:10.1186/s12245-015-0088-x) contains supplementary material, which is available to authorized users.

## Background

The increasing burden of trauma and non-communicable diseases in low- and middle-income countries (LMICs) has emphasized the need for effective emergency care to alleviate the morbidity and mortality associated with acute illness and injury [1–4]. This has led international organizations, including the World Bank, World Health Organization, and United Nations Children’s Fund, to place substantial emphasis on the development and strengthening of systems of emergency care in resource-limited settings [4–6].

As emergency care in LMICs expands, there is a growing need to measure and improve the quality and safety of this care. Well-developed quality assurance systems currently exist in high-income countries [7, 8], where the development and use of quality indicators has led to major improvements in the standard of emergency care provided [9]. While systematic performance measurement is the foundation of quality health care [10], quality and safety indicators used in developed countries may not be appropriate in resource-limited settings [9, 11]. Indeed, little is known about the metrics being used to measure emergency care in LMICs, and to our knowledge, no study has cataloged which metrics are being used. This limits the ability of emergency departments (EDs) in low-resource settings to implement quality assurance programs.

The objective of this systematic review is to catalog and classify existing performance metrics that have been used to measure the quality of ED care in resource-limited settings.

## Methods

### Search strategy

A medical librarian searched MEDLINE, Embase, and CINAHL from the earliest available date to September 30, 2013. The following search terms were used: quality, quality assurance, quality indicators, utilization review, combined with any of the following: emergency, emergency medical services, emergency service, accident and emergency, emergency department, and any of the following: developing countries, third world, and resource limited (or low or poor). The search was restricted to English language articles.

The gray literature was searched through an internet-based search of the websites of relevant international and emergency medicine organizations such as the World Bank, International Federation for Emergency Medicine, International Medical Corps and United Nations, as well as a Google search using combinations of the following terms: quality, quality assurance, quality indicators (or measures), performance indicators (or measures), safety indicators (or measures), combined with any of the following: emergency, emergency medicine, emergency medical care, emergency services, emergency unit (or department), and any of the following: developing, resource-limited, and low- or middle-income countries.

### Article selection

Studies were eligible for inclusion if they were conducted in a low- or middle-income country, as defined by the World Bank classification system [12], and addressed quality markers, indicators, or metrics for care in an ED or emergency unit. Studies conducted in multiple countries were included if one or more of the countries was a LMIC. A metric was defined as a performance measure that assessed a predefined quality standard. If an article analyzed the quality of care in the hospital as a whole, including the ED, the article was included only if the article separately reported metrics measured in the ED. Studies of prehospital care, emergency obstetrics, and secondary injury prevention were excluded. If a study included both prehospital and in-hospital metrics, it was included only if the in-hospital metrics were separately listed.

Articles were excluded if they were opinions or review articles that did not feature original data. Articles that described potential indicators, but did not implement them or measure them, were also excluded, as the focus of the present review was on indicators that have been previously utilized.

One author (ELA) initially reviewed the titles and abstracts of all articles identified by the search terms to exclude all clearly ineligible articles. The remaining titles and abstracts were re-reviewed by two authors (ELA and SAR), and a consensus was reached to create a list of potentially relevant articles. The full text articles were then reviewed by three authors (ELA, SAR, and RHM) to confirm eligibility. Given the limited literature on this topic, articles were not excluded based on quality of the study or publication.

### Data extraction and analysis

Three authors (ELA, SAR, and RHM) reviewed the full text of all relevant articles using a standardized form to extract individual quality metrics and study details. If a study examined both ED and hospital care, only the ED metrics were included. However, if a study looked exclusively at care within the ED, but included metrics or outcomes that occurred after the ED stay, such as mortality, these metrics were included.

For ease of comparison, certain structural metrics were collapsed into predefined categories. For example, metrics examining availability of specific medications were combined into a single metric by predefined medication class.

Once extracted, each metric was categorized by a predefined matrix based on the IOM framework of healthcare quality (Table 1) [13]. These were then further classified into the Donabedian framework of health care consisting of causally linked and measurable categories (Table 2) [14].

**Table 1:** The Institute of Medicine framework of healthcare quality

Safety	Avoiding injuries to patients from the care that is intended to help them
Effective	Providing services based on scientific knowledge to all who could benefit and refraining from providing services to those not likely to benefit
Patient-centered	Providing care that is respectful and responsive to individual patient preferences, needs, and values
Timely	Reducing waits and sometimes harmful delays for both those who receive and those who give care
Efficient	Avoiding waste, including waste of equipment, supplies, ideas, and energy
Equitable	Providing care that does not vary in quality because of personal characteristics such as gender, ethnicity, geographic location, and socioeconomic status

**Table 2:** Donabedian framework of health care

Structure	The human, physical, and financial resources available to provide health care
Process	The care or health service provided to the patient
Outcome	The resulting effect on the health of the patient or population

Each quality metric was assigned to only one domain. A fourth author (JDS) then independently reviewed and classified the extracted quality metrics, with a weighted kappa of 0.89.

### Articles applying the WHO/IATSIC guidelines

Several studies used the indicators in the World Health Organization (WHO) and International Association for Trauma and Intensive Care (IATSIC) Guidelines for Essential Trauma Care. These guidelines offer a toolkit of over 200 metrics for the internal assessment of trauma care at the hospital level, focused on human resources (staffing and training) and physical resources (infrastructure, equipment, and supplies). While the guidelines reference emergency care, they are intended to assess specifically trauma capacity of a hospital as a whole, which was not the present focus of this review. A number of studies have been performed applying the WHO/IATSIC indicators, and thus including them in the primary analysis would have disproportionately weighted the indicators of this study. Therefore, quality indicators found in this group of articles were examined separately. Only reported metrics from each study were extracted.

## Results

**Fig. 1:**
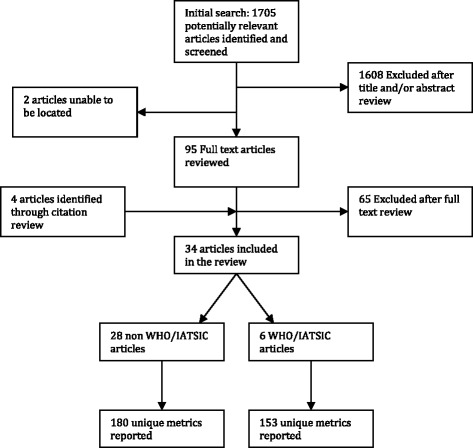
Process of inclusion of studies in the systematic literature review

The literature search identified 1705 titles (Fig. 1). Of these, 97 were eligible for full text review. Two articles could not be located, after exhaustive search by a trained medical librarian. Of the 95 reviewed, 30 met inclusion criteria. The references of included articles were also reviewed, yielding an additional 4 articles for inclusion. In total, 34 articles were included, 6 of which reported the implementation of the WHO/IATSIC guidelines [15–49]. The summary characteristics of the non-WHO/IATSIC included articles are listed in Table 3. Detailed descriptions of each article included are listed in the Additional file 1.

**Table 3:** Summary characteristics of articles (each country is counted individually in multinational studies)

	Number of articles
Country income level	
Low-income	7
Low-middle income	20
Upper-middle income	17
Region	
Europe and Central Asia	2
Middle East and North Africa	3
East Asia and Pacific	8
Latin America and the Caribbean	8
South Asia	11
Sub-Saharan Africa	12

Excluding the WHO/IATSIC articles that were analyzed separately, 180 quality metrics were extracted from the remaining 28 articles, including 129 unique indicators. The majority of all reported measures were not disease-specific (*n* = 126; 70 %) but focused on metrics that applied to patients with a variety of diseases. The 54 measures that were disease-specific focused on illnesses related to the following: respiratory (*n* = 23), systemic states (*n* = 9), hematologic (*n* = 7), circulatory and cardiovascular (*n* = 6), neurologic (*n* = 4), trauma (*n* = 2), endocrine, metabolic and nutritional disease (*n* = 1), fluid and electrolyte disorders (*n* **=** 1), and gastrointestinal diseases (*n* **=** 1).

Most metrics were process (*n* = 102; 57 %) and structure measures (*n* = 49; 27 %). Only 16 % (*n* = 29) were related to outcomes (Table 4). Regarding the IOM domains, most metrics evaluated the effectiveness of care (*n* = 94; 52 %). These were predominantly markers of effective processes, such as adherence to a full physical exam and appropriate test ordering, or of effective structures such as the availability of essential supplies. A small number dealt with the effectiveness of outcomes, such as mortality. Metrics assessing timeliness of care (*n* = 51; 28 %) dealt primarily with processes such as time to provider and outcomes such as length of stay. Few metrics addressed patient-centered care (*n* = 20; 11 %); those that did looked primarily at patient satisfaction. Seven percent (*n* = 14) of metrics addressed the safety of care and focused on complications of care and the appropriate use of medications. Resource-efficient (*n* = 5; 3 %) and equitable (*n* = 2; 1 %) measures were rare.

**Table 4:** Frequency of indicators extracted from non-WHO/IATSIC studies, classified by Donabedian and Institute of Medicine domains

	Structure	Process	Outcome	Total
	*n* (% of total)	*n* (% of total)	*n* (% of total)	*n* (% of total)
Effective	39 (22 %)	48 (27 %)	7 (4 %)	94 (52 %)
Patient-centered	4 (2 %)	3 (2 %)	13 (7 %)	20 (11 %)
Timely	4 (2 %)	40 (22 %)	7 (4 %)	51 (28 %)
Safe	1 (1 %)	5 (3 %)	2 (1 %)	8 (4 %)
Efficient	0 (0 %)	5 (3 %)	0 (0 %)	5 (3 %)
Equitable	1 (1 %)	1 (1 %)	0 (0 %)	2 (1 %)
Total	49 (27 %)	102 (57 %)	29 (16 %)	180 (100 %)

Among the articles that implemented the WHO/IATSIC surveys, 336 metrics were extracted. Many of these were repeated within articles; a total of 153 unique metrics were identified. The majority of the metrics were related to the effectiveness of the structure of care (*n* = 141; 92 %). The remaining metrics dealt with safe structure (*n* = 7; 5 %), safe process (*n* = 4; 2 %), and efficient process (*n* = 1; 1 %).

## Discussion

The EM quality literature provides strong evidence that quality improvement programs can improve quality of care and patient outcomes [7, 8, 49]. Understanding that improvement requires measurement [7, 8], the availability of applicable measures for emergency care in LMICs is essential. While there has been dramatic growth in the delivery of emergency care in LMICs over the last decade, little is known about the quality of care or how to evaluate it.

Through a rigorous search strategy and structured data extraction, this systematic review collected and analyzed published ED quality metrics in LMICs. Our data show that only a limited number of metrics have been reported, the majority of which focus on structures or processes of care, rather than on patient outcomes. The limited metrics suggest a pressing need to develop and implement performance measures that reflect the spectrum of emergency care in LMICs.

Our study shows that when applying a structured framework for quality metrics to the over 150 metrics currently used to measure the quality of emergency care in LMICs, these metrics do not achieve balance. The majority of these metrics are focused on process and structure, likely reflecting the greater availability of data in these domains. Process metrics, making up over half of all metrics reported in our study, were predominantly centered on operational measures looking at the effectiveness and timeliness of ED processes. Although literature in high-income countries suggests that the most successful performance measures for quality improvement are outcome metrics related to ED time intervals (length of stay, arrival to assessment/admission) and patient centeredness (72-h ED returns, patients who left without being seen) [8, 50, 51], we found that only 16 % of metrics in LMICS were outcome based. Even fewer (11 %) were patient-centered. Future work is needed to analyze why patient-centered and outcome indicators have not yet been implemented and to develop contextually appropriate measures.

It is interesting to note that few resource-efficient metrics were reported. EDs in LMICs are increasingly facing the pressures of high patient volumes, limited resources, and an ever-high burden of disease [52]. Given this landscape, it is essential that the care being delivered is resource-efficient and high quality. Unlike in high-income settings, where individual providers typically do not face resource constraints, in a low-resource setting, the use of an expensive medication or lab test on one patient may consume the resources needed to treat the next. The limited resources and significant unmet need for health care in LMICs make it essential that care is both efficient and equitable. The ability to provide more with less is inherently tied to the ability to create streamlined processes and efficiency in both operations and supply-chain management. The WHO has noted that health systems in low-income countries have the greatest potential for increasing efficiency with minimal investment [53, 54]. Our study suggests the need to identify metrics that can measure these efficiencies and contribute to their improvement.

Prior efforts have been made to identify feasible quality metrics in emergency care. In addition to metrics which came out of the International Federation of Emergency Medicine’s Symposium for Quality and Safety, an expert consensus study conducted in South Africa identified 58 performance indicators that they deemed to be feasible to measure in low-resource settings (37 structure based, 20 process based, and 1 outcome based) [52, 55]. Our study shows that few of these are being reported currently. Only 20 of the proposed measures (34 %) were identified in our review (11 structural measures, 4 process measures in the non-WHO/IATSIC studies, and an additional 5 structural measures in the WHO/IATSIC studies). This suggests that there are a large number of potentially feasible quality indicators that are not being studied in LMICs. Future research should examine the reasons for this discrepancy and either modify these metrics or support their implementation as needed.

While the metrics identified in this review have all been successfully measured in LMICs, the ease of measurement is not compared or documented. The well-documented barriers that exist to measure these metrics in developed countries are likely more significant in LMICs. Lack of senior management with strong commitment or training in quality improvement methods, limited resources to collect and analyze data, and a lack of clarity around which metrics are most important have all been noted to limit institutional abilities to effectively measure the quality of care [7]. These challenges are magnified in LMICs, particularly within EDs, as emergency medicine is in its nascency in many countries.

### Limitations

The study was limited to English language articles and may have missed metrics reported in the non-English language literature. Although our search terms were broad, there may be articles using different terms that we did not capture. Similarly, it will not capture unpublished metrics currently in use. There are no standardized definitions for the classification of metrics in the IOM and Donabedian domains, resulting in a degree of subjectivity in their classification. To address this, three authors reached consensus on each metric, and a fourth blinded reviewer re-classified the metrics with a kappa that was robust. Finally, the IOM and Donabedian domains were developed for high-resource settings, and their applicability to a lower-resource setting is unknown.

## Conclusions

As emergency medicine continues to grow as a field in LMICs, there is an increasing need for effective metrics to measure the quality of this care. This systematic review of performance measures suggests that although there are a number of published quality metrics currently used to assess emergency care in LMICs, these do not adequately assess all aspects of emergency care. This study has demonstrated that broad metrics have been applied in LMICs, however identified the need for the development of more comprehensive measures that are locally applicable. As metrics for LMICs are developed, they must be implemented and then reported on to develop global standards of quality measurement in emergency care.

## Additional file

Additional file 1:
**Appendix 1.** Included Articles (non WHO/IATSIC). **Appendix 2.** Included Articles (WHO/IATSIC). (DOCX 96.9 kb)

## References

[1] Hofman K, Primack A, Keusch G, Hrynkow S (2005). Addressing the growing burden of trauma and injury in low- and middle-income countries. Am J Public Health.

[2] Murray CJ, Lopez AD (1996). The global burden of disease: a comprehensive assessment of mortality and disability from diseases, injuries, and risk factors in 1990 and projected to 2020.

[3] Murray CJL, Lopez AD (1996). Global health statistics: a compendium of incidence prevalence and mortality estimates for over 200 conditions.

[4] Anderson P, Petrino R, Halpern P, Tintinalli J (2006). The globalization of emergency medicine and its importance for public health. Bull World Health Organ.

[5] World Bank (1995). Minimum package of health services: criteria, method and data.

[6] Gove S (1997). Integrated management of childhood illness by outpatient health workers: technical basis and overview. Bull World Health Organ.

[7] Graff L, Stevens C, Spaite D, Foody J (2002). Measuring and improving quality in emergency medicine. Acad Emerg Med.

[8] Sørup CM, Jacobsen P, Folberg JL (2013). Evaluation of emergency department performance—a systematic review on recommended performance and quality-in-care measures. Scand J Trauma Resusc Emerg Med.

[9] Lindsay P, Schull M, Bronskill S, Anderson G (2002). The development of indicators to measure the quality of clinical care in emergency departments following a modified-Delphi approach. Acad Emerg Med.

[10] Scott KW, Jha AK (2014). Putting quality on the global health agenda. N Engl J Med.

[11] Beattie E, Mackway-Jones K (2004). A Delphi study to identify performance indicators for emergency medicine. Emerg Med J.

[12] The World Bank. World Bank List of Economies. 2013. Accessed January 8, 2014; Available from: http://data.worldbank.org/about/country-classifications/country-and-lending-groups

[13] Institute of Medicine (2001). Crossing the quality chasm: a new health system for the twenty-first century.

[14] Donabedian A (1996). Evaluating the quality of medical care. Milbank Mem Fund Q.

[15] Achan J, Tibenderana J, Kyabayinze D, Mawejje H, Mugizi R, Mpeka B (2011). Case management of severe malaria—a forgotten practice: experiences from health facilities in Uganda. PLos One.

[16] Adamu A, Maigatari M, Lawal K, Iliyasu M (2010). Waiting time for emergency abdominal surgery in Zaria, Nigeria. Afr Health Sci.

[17] Akoglu S, Topacoglu H, Karcioglu O, Cimrin AH (2004). Do the residents in the emergency department appropriately manage patients with acute asthma attach? A study of self-criticism. Adv Ther.

[18] Borlina LP, Silva EL C e, Ghislandi G, Timi JRR (2010). Emergency-room doctors’ knowledge about oral anticoagulants and its management. J Vasc Bras.

[19] Chadha R, Singh A, Kalra J (2012). Lean and queuing integration for the transformation of health care processes. Clin Gov.

[20] Cinar O, Turkan H, Duzok E, Sener S, Uzun A, Durusu M (2010). Do we know how to use oxygen properly in the emergency department. J Clin Anal Med.

[21] Goel A, Kumar S, Bagga M (2004). Epidemiological and Trauma Injury and Severity Score (TRISS) analysis of trauma patients at a tertiary care centre in India. Natl Med J India.

[22] Hashami Z, Haider A, Zafar SN, Kisat M, Moosa A, Siddiqui F (2013). Hospital-based trauma quality improvement initiatives: first step toward improving trauma outcomes in the developing world. J Trauma Acute Care Surg.

[23] Idro R, Aloyo J (2004). Manifestations, quality of emergency care and outcome of severe malaria in Mulago Hospital, Uganda. Afr Health Sci.

[24] Jalili M, Shalileh K, Mojtahed A, Mojtahed M, Moradi-Lakeh M (2012). Identifying causes of laboratory turnaround time delay in the emergency department. Arch Iran Med.

[25] Kirenga JB, Okot-Nwang M (2012). The proportion of asthma and patterns of asthma medications prescriptions among adult patients in the chest, accident and emergency units of a tertiary health care facility in Uganda. Afr Health Sci.

[26] Loch A, Twin T, Zakaria IM, Abidin I, Ahmad WA, Hautmann O (2013). Failure to improve door-to-needle time by switching to emergency physician-initiated thrombolysis for ST elevation myocardial infarction. Postgrad Med J.

[27] Salleh FM, Fathil SM, Ahmad Z, Che’Man Z (2010). Early goal-directed therapy in the management of severe sepsis/septic shock in an academic emergency department in Malaysia. Crit Care Shock.

[28] Nayeri ND, Aghajani M (2012). Patients’ privacy and satisfaction in the emergency department: a descriptive analytical study. Nurs Ethics.

[29] Nguyen HB, Kuan WS, Batech M, Shrikhande P, Mahadevan M, Li CH (2011). Outcome effectiveness of the severe sepsis resuscitation bundle with addition of lactate clearance as a bundle item: a multi-national evaluation. Crit Care.

[30] Nolan T, Angos P, Cunha AJ, Muhe L, Qazi S, Simoes EA (2001). Quality of hospital care for seriously ill children in less-developed countries. Lancet.

[31] Oliveira AC, Marziale MH, Paiva MH, Lopes AC (2009). Knowledge and attitude regarding standard precautions in a Brazilian public emergency service: a cross-sectional study. Rev Esc Enferm USP.

[32] Onwukike M, Olaloye OA, Oni OO (2001). Teaching hospital perspective of the quality of trauma care in Lagos, Nigeria. World J Surg.

[33] Parekh K, Russ S, Amsalem DA, Rambaran N, Wright SW (2013). Who leaves the emergency department without being seen? A public hospital experience in Georgetown, Guyana. BMC Emerg Med.

[34] Payal P, Sonu G, Anil GK, Prachi V (2013). Management of polytrauma patients in emergency department: an experience of a tertiary care health institution of northern India. World J Emerg Med.

[35] Razzak JA, Hyder AA, Akhtar T, Khan M, Khan UR (2008). Assessing emergency medical care in low income countries: a pilot study from Pakistan. BMC Emerg Med.

[36] Rauf W, Blitz JJ, Geyser MM, Rauf A (2008). Quality improvement cycles that reduced waiting times at Tshwane District Hospital Emergency Department. SA Fam Pract.

[37] Rehmani R (2004). Emergency section and overcrowding in a University Hospital of Karachi, Pakistan. J Pak Med Assoc.

[38] Shahid M, Hameed K, Iqbal R, Afzal O, Nakeer R, Razzak J (2012). Accuracy of diagnosis and relationship with quality of emergency medicine training program. J Coll Physicians Surg Pak.

[39] Sultana A, Riaz R, Hameed S, Syed Arshad S, Iffat T, Arshia B (2010). Patient satisfaction in emergency department of District Head Quarters Hospital, Rawalpindi. Rawal Med J.

[40] Tamburini G, Di Mario S, Maggi RS, Vilariam JN, Grove S (1999). Evaluation of guidelines for emergency triage assessment and treatment in developing countries. Arch Dis Child.

[41] Waxman MH, Kimaiyo S, Ongaro N, Wools-Kaloustian KK, Fanigan TP, Carter EJ (2007). Initial outcomes of an emergency department rapid HIV testing program in western Kenya. AIDS Patient Care STDS.

[42] Ye L, Zhou G, He X, Shen W, Gan J, Zhang M (2012). Prolonged length of stay in the emergency department in high-acuity patients at a Chinese tertiary hospital. Emerg Med Australasia.

[43] Aboutanos MB, Mora F, Rodas E, Salamea J, Parra MO, Salgado E (2010). Ratification of IATSIC/WHO’s guidelines for essential trauma care assessment in the South American region. World J Surg.

[44] Arreola-Risa C, Mock C, Vega Rivera F, Romero Hicks E, Guzmán Solana F, Porras Ramírez G (2006). Evaluating trauma care capabilities in Mexico with the World Health Organization’s Guidelines for Essential Trauma Care publication. Rev Panam Salud Publica.

[45] Hanche-Olsen TP, Alemu L, Viste A, Wisborg T, Hansen KS (2012). Trauma care in Africa: a status report from Botswana, guided by the World Health Organization’s “Guidelines for Essential Trauma Care.”. World J Surg.

[46] Mock C, Nguyen S, Quansah R, Arreola-Risa C, Viradia R, Joshipura M (2006). Evaluation of trauma care capabilities in four countries using the WHO-IATSIC guidelines for essential trauma care. World J Surg.

[47] Son NT, Mock C (2006). Improvements in trauma care capabilities in Vietnam through use of the WHO-IATSIC guidelines for essential trauma care. Int J Inj Contr Saf Promot.

[48] Tachfouti N, Bhatti JA, Nejjari C, Kanjaa N, Salmi LR (2011). Emergency trauma care for severe injuries in a Moroccan Region: conformance to French and World Health Organization Standards. J Healthc Qual.

[49] Molyneux E, Ahmad S, Robertson A (2006). Improved triage and emergency care for children reduces inpatient mortality in a resource-constrained setting. Bull World Health Organ.

[50] Alessandrini EA, Knapp J (2011). Measuring quality in pediatric emergency care. Clin Ped Emerg Med.

[51] Beniuk K, Boyle AA, Clarkson PJ (2012). Emergency department crowding: prioritising quantified crowding measures using a Delphi study. Emerg Med J.

[52] Maritz D, Hodkinson P, Wallis L (2010). Identification of performance indicators for emergency centres in South Africa: results of a Delphi study. Int J Emerg Med.

[53] Evans DB, Tandon A, Murray CJ, Lauer JA (2001). Comparative efficiency of national health systems: cross national econometric analysis. BMJ.

[54] Kruk ME, Freedman LP (2008). Assessing health system performance in developing countries: a review of the literature. Health Policy.

[55] Lecky F, Benger J, Mason S, Cameron P, Walsh C (2014). The International Federation for Emergency Medicine framework for quality and safety in the emergency department. Emerg Med J.

