# Supplementary material for: Emergency department quality and safety indicators in resource-limited settings: an environmental survey
Source: Int J Emerg Med. 2015 Oct 31;8:39. doi: 10.1186/s12245-015-0088-x (PMC4628609; doi:10.1186/s12245-015-0088-x)
Supplement: Additional file 1: — Appendix 1. Included Articles (non WHO/IATSIC). Appendix 2. Included Articles (WHO/IATSIC). (DOCX 96.9 kb) [file 12245_2015_88_MOESM1_ESM.docx]

Appendix 1: Included Articles (non WHO/IATSIC)

| Author/Year | Setting | Design | Objective | Disease Topic | Subjects | N | Domains identified and example measures |
| --- | --- | --- | --- | --- | --- | --- | --- |
| Achan, 2011 | Multiple hospitals, **Uganda** | Cross sectional survey | To evaluate the management of severe malaria | Malaria | Randomly selected health facilities | 105 health facilities in 11 districts over three month period | Structure-effective (presence of triage), process-effective (was history recorded), process-timely (time to provider), outcome-patient centered (patient satisfaction) |
| Adamu, 2010 | University hospital, **Nigeria** | Prospective cohort | To determine waiting time between presentation at hospital and operative intervention in patients with acute abdominal complaints | Acute abdominal disease | Patients with acute abdominal disease requiring surgery | 488 patients presenting to the ED over a 24 month period | Process-timely (time to provider) |
| Akoglu, 2004 | University hospital; **Turkey** | Prospective cohort | Investigate resident management and use of protocols and guidelines when treating patients with asthma attacks | Asthma | Patients presenting with asthma and admitted over 12 month period | 72 patients | Processes-effective (was exam recorded); process-efficient (were systemic steroids given PO instead of IV) |
| Borlina, 2010 | Public and private hospitals, **Brazil** | Cross-sectional questionnaire | Evaluate physician’s knowledge of oral anticoagulation and drug interactions; determine if this knowledge is integrated into routine use | N/A | EM physicians at two public and three private hospitals | 100 EM physicians | Process-safe (provider asks about anticoagulation use), structure-effective (provider knowledge of anticoagulants) |
| Chadha, 2012 | Mission hospital, **India** | Descriptive analytical study | To report the implementation of a lean health care model to improve the performance of the health care system in an ED | N/A | One hospital’s Emergency Department | N/A | Outcome-timely (ED LOS), process=timely (time to provider) |
| Cinar, 2010 | University hospitals, **Turkey** | Cross-sectional questionnaire | To determine ED healthcare workers knowledge of oxygen therapy | Respiratory | ED healthcare workers (Nurses, doctors, interns) | 100 healthcare workers | Structure-effective (knowledge of airway/breathing skills) |
| Goel, 2004 | Academic, urban tertiary hospital, **India** | Prospective observational study | To obtain the epidemiological profile of Indian trauma patient | Trauma | All trauma patients admitted to the surgery service | 180 patients | Outcome-effective (unexpected death) |
| Hashmi, 2013 | University teaching hospital, **Pakistan** | Pre/post | To determine the impact of a hospital based trauma team | All | Trauma patients in the trauma database over 12 year period | 1227 patients | Outcome-effective (mortality), outcome-safe (complications) |
| Idro, 2004 | Urban, academic hospital; **Uganda** | Prospective cohort | Describe the quality of care children with severe malaria | Malaria | Children with severe malaria over 2 month period | 784 children | Structure-effective (are all essential drugs available); process-timely (time to first dose of quinine); process-effective (renal function measured); outcome-effective (mortality); outcome-patient centered (patient satisfaction) |
| Jalili, 2012 | University teaching hospital, **Iran** | Cross-sectional study | To evaluate laboratory turn around time an ED and to generate a simple model for identifying the primary causes for delay | N/A | Laboratory tests over a one week period | 435 tests | Process-timely (lab turn around time) |
| Kirenga, 2012 | University hospital, **Uganda** | Retrospective chart review | To determine the proportion of asthmatics receiving recommended therapy | Asthma | All asthma patients in the ED and Chest Clinic over 12 month period | 1600 (ED), 792 (Chest) patients over 12 month period | Process-effective (adherence to clinical guidelines), process-efficient (inappropriate antibiotic use) |
| Loch, 2013 | University teaching hospital, **Malaysia** | Pre/post | To assess the effect of transferring responsibility for thrombolysis to the ED doctors on door-to-needle times and to identify predictors of prolonged times | STEMI | Patients with STEMI given thrombolysis over two study periods (15 months and 9 months) | 297 patients | Process-timely (door to needle), process-safe (inappropriate triage), process-effective (incorrect ECH interpretation) |
| Salleh, 2010 | University teaching hospital, **Malaysia** | Prospective cohort | Evaluate implementation of Early Goal Directed Therapy | Sepsis | ED patients with severe sepsis over three month period | 16 patients | Process-timely (all early goal directed therapy goals achieved in 6 hours), outcome-timely (ED LOS) |
| Nayeri, 2010 | University hospital, **Iran** | Descriptive analytical study | To investigate the extent to which patient privacy is observed and its correlation with patient satisfaction | All | 360 patients admitted to the ED over 3 months | 360 patients | Outcome-patient centered (patient satisfaction) |
| Nguyen, 2011 | Multiple tertiary care hospitals; **China, India, Korea, Singapore, Taiwan** | Prospective cohort study | Examine the compliance and effectiveness of surviving sepsis campaign resuscitation bundle with addition of lactate clearance | Sepsis | ED patients in 8 EDs over 18 months who met criteria for surviving sepsis campaign bundle | 556 patients | Process-effective (compliance with sepsis bundle); outcome-effective (mortality) |
| Nolan, 2001 | District and teaching hospitals; **Bangladesh, Dominican Republic, Ethiopia, Indonesia, Philippines, Tanzania, Uganda** | Cross-sectional, observational study; using qualitative survey and structured observation. | To assess the quality of priority screening (triage), ED diagnosis and care; to determine the impact of improved triage for sick children | All | 13 district hospitals and 8 teaching hospitals in 7 countries | 21 hospitals | Structure-effective (presence of formal triage algorithm); process-effective (quality of triage); process-timely (delays in treatment), process-efficient (inappropriate treatment given) |
| Oliveira, 2009 | Government hospital, **Brazil** | Cross-sectional survey | To assess the knowledge and attitude of health care professionals towards universal precaution; determine the rates of occupational accidents involving biological substances | All | Health care workers in the ED over six month period | 238 health care workers | Process-safe (completion of accident report), structure-safe (knowledge of safety), outcome-safe (rate of occupational exposure) |
| Onwudike, 2001 | Academic hospital; **Nigeria** | Prospective observational | To determine the quality of trauma care using the trauma and Injury severity score | All | Injured patients admitted over a 9 month period | 253 patients | Outcome-effective (unexpected death); process-timely (those with ISS>15 seen within 30 minutes) |
| Parekh, 2013 | Public hospital, **Guyana** | Retrospective cross-sectional analysis | To determine the left without being seen rate, and identify factors that might influence it | All | ED patients over a two week period | 3027 visits | Outcome-timely (left without being seen) |
| Payal, 2013 | University hospital, **India** | Retrospective cross sectional study | To elicit complaints, suggestions and possible solutions for the management of patients with poly trauma | Trauma | Polytrauma patients over a two month period | 210 patients | Process-timely (time to treatment plan in ED) |
| Razzak, 2008 | Multiple hospitals and health facilities **Pakistan** | Cross sectional assessment | To assess the availability and quality of facility based emergency medical care in the government health system | All | Community leaders, health care providers and health care facility administrators | 22 rural and 20 urban health care facilities | Structure-effective (availability of medication), structure-timely (accessibility of facility after working hours), structure-equitable (facility distance from a major road), structure-effective (percent of patients seen by a physician), outcome-patient centered (satisfaction of community leaders with emergency care) |
| Rauf, 2008 | District hospital, **South Africa** | Pre/post | To analyze the problems, and measure the effect of solutions implemented, on ED waiting times | All | ED patients over two four month periods | 150 patients | Process-timely (time to provider) |
| Rehmani, 2004 | University hospital; **Pakistan** | Retrospective chart review | To quantify the extent of ED overcrowding and identify solutions | All | All ED patients with >6 hour length of stay (LOS) in the ED during a three month period | 9630 patients | Outcome-timely (ED LOS), structure-timely (inpatient bed ready), process-timely (wait time for labs), process-equitable (LOS influenced by patient financial constraints) |
| Shahid, 2012 | University hospital; **Pakistan** | Retrospective chart review | To determine the accuracy of ED diagnosis | All | 3 years of admitted ED patients | 8488 charts | Outcome-effective (accuracy of diagnosis by congruence with ER diagnosis and diagnosis at time of hospital discharge) |
| Sultana, 2010 | Unspecified; **Pakistan** | Cross sectional descriptive study | To determine attitudes and behaviors of healthcare providers through survey of patients and providers | All | Patients and providers at one ED over 2 week period | 500 patients | Structure-patient centered (phone available); process-patient centered (satisfied with behavior health care providers); outcome-patient centered (patient satisfaction); structure-timely (investigations available in-hospital); process-timely (time to provider); outcome-timely (ED length of stay) |
| Tamburlini, 1999 | 600 bed hospital; **Brazil** | Prospective cohort | To evaluate performance of a simplified triage score (ETAT) for children | All | All infants presenting to the ED over 10 week period | 3837 patients | Process-effective (number of admitted patient identified by triage system); process-timely (time to nurse initiated emergency treatments) |
| Waxman, 2007 | University hospital, **Kenya** | Retrospective chart review | To evaluate the effectiveness of an ED HIV program | HIV | ED patients with signs of symptoms of HIV over 8 months | 1371 patients | Process-effective (follow up visit for HIB after ED initiated testing program), process-timely (1 month follow up) |
| Ye, 2012 | University hospital, **China** | Retrospective chart review | To investigate reasons high acuity patients have with prolonged ED LOS | High acuity patients | High-acuity ED patients over a 12 month period | 7,966 patients | Outcome-timely (ED LOS), process-timely (boarding < 2 hours) |

Appendix 2. Included Articles (WHO/IATSIC)

| Author/Year | Setting | Design | Objective | Disease Topic | Subjects | N | Domains identified and example measures |
| --- | --- | --- | --- | --- | --- | --- | --- |
| Aboutanos, 2010 | Multiple Hospitals; **Ecuador** | Cross Sectional …? | Evaluate the usefulness of the IATSIC/WHO  Guidelines for Essential  Trauma Care in South America | Trauma | Hospitals | 24 hospitals: 5 large hospitals, 15 small hospitals, 4 basic hospitals | Process-efficient (trauma registry), process-safe (QI programs), structure-effective (availability of supplies), Structure-safe (sharps disposal) |
| Arreola-Risa, 2006 | Multiple Hospitals; **Mexico** | Cross Sectional | Identify affordable, sustainable methods to strengthen trauma care capabilities  in Mexico, using the IATSIC/WHO  Guidelines for Essential  Trauma Care | Trauma | Government owner or operated healthcare facilities | 16 healthcare facilities: 5 clinics, 4 small hospitals, 7 large hospitals | Process-efficient (trauma registry), process-safe (QI programs), structure-effective (availability of supplies), structure-safe (sharps disposal ) |
| Hanche-Olsen, 2012 | Multiple Hospitals; **Botswana** | Cross Sectional | Evaluate the trauma care capabilities of Botswana, using the IATSIC/WHO  Guidelines for Essential  Trauma Care | Trauma | All government hospitals | 27 government hospitals: 16 primary, 9 district, 2 referral | Structure-effective (availability of supplies) |
| Mock, 2006 | Multiple Hospitals; **Mexico, Vietnam, India, Ghana** | Cross Sectional | Identify affordable and sustainable methods to strengthen trauma care using the IATSIC/WHO  Guidelines for Essential  Trauma Care | Trauma | Healthcare facilities | 100 healthcare facilities: 51 rural clinics, 34 small hospitals, 15 large hospitals | Process-efficient (trauma registry), process-safe (QI programs), structure-effective (availability of supplies), structure-safe (sharps disposal) |
| Son, 2006 | Multiple Hospitals; **Vietnam** | Cross Sectional | Assess trauma care in Vietnam using the IATSIC/WHO  Guidelines for Essential  Trauma Care | Trauma | Healthcare facilities | 23 healthcare institutions: 12 rural clinics, 6 district hospitals, 4 city hospitals, 1 emergency transport station | Structure-effective (availability of supplies) |
| Tachfouti, 2010 | University teaching hospital **Morocco** | Cross Sectional | Assess actual structure and processes of emergency trauma care in Morocco using the IATSIC/WHO  Guidelines for Essential  Trauma Care | Trauma | Hospital | 1 university teaching hospital | Process-efficient (trauma registry), process-safe (QI programs), structure-effective (availability of supplies ) |
